# Baker’s Cyst on F-18 FDG PET/CT in a Patient with Ocular Melanoma

**DOI:** 10.4274/Mirt.14

**Published:** 2012-08-01

**Authors:** Hakan Cayvarlı, Erkan Derebek

**Affiliations:** 1 Dokuz Eylül University Medical Faculty, Department of Nuclear Medicine, İzmir, Turkey

**Keywords:** Fluorodeoxyglucose F18, Positron emission tomography, popliteal cyst

## Abstract

We present the case of a 56-year-old man with the clinical diagnosis of ocular melanoma. As a part of staging, he underwent an F-18 FDG PET/CT scan for investigating distant metastasis. On PET scan, an increased focal area of F-18 FDG uptake was seen behind patient's right knee that can be confused with distant metastasis. On CT scan, there was a fluid density in the same location. The probable diagnosis was Baker’s cyst. Later, USG and MRI confirmed this diagnosis.

**Conflict of interest:**None declared.

## INTRODUCTION

PET/CT is a challenging imaging modality in oncology and it can be used in diagnosing, staging, restaging and evaluating response to therapy. There are numerous reports in the literature about PET/CT interpretation pitfalls. In this case, we report a Baker’s cyst in a patient with ocular melanoma, accumulating F-18 FDG on PET scan, that can be confusing by mimicking distant metastasis.

## CASE REPORT

**Figure 1:**
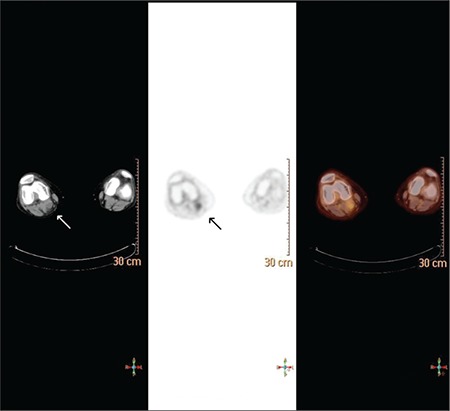
Axial slices of F-18 FDG PET/CT study showing an increased focal uptake behind patient’s right knee on PET scan (SUVmax 3.2) (black arrow). A fluid density on CT scan in the same location compatible with Baker’s Cyst (white arrow)

A 56-year-old man was referred to an opthalmologist with a complaint of progressive vision loss in his left eye for 4 months. After clinical evaluation, the diagnosis was ocular melanoma. As a part of staging, he underwent an F-18 FDG PET/CT scan for investigating distant metastasis. In the PET/CT study, sixty minutes after an intravenous injection of 11.1 mCi F-18 fluorodeoxyglucose (FDG) with the patient fasting over 8 hours, an increased focal area of F-18 FDG uptake was seen behind patient’s right knee on PET scan (SUVmax 3.2) (Figure 1). On CT scan, there was a fluid density in the same location. The probable diagnosis was Baker’s cyst for this finding. Later, USG and MRI confirmed this diagnosis. 

## LITERATURE REVIEW AND DISCUSSION

Baker’s cyst represents a fluid distention of a bursa between the gastrocnemius and semimembranosus tendons through a communication with the knee joint (1,2). Baker’s cyst can appear clinically as a posterior knee mass, mimicking a true soft tissue mass (2). The clinical presentations of Baker’s cyst are; local pain, swelling, posterior knee tightness feeling on walking or activity and a palpable mass along the medial side of the popliteal fossa (2). In adults, Baker’s cyst may be caused by an inflammatory joint disease or mechanical intra-articular derangements of the knee joint (3). The most frequent associated arthropathy of Baker’s cyst is osteoarthritis (50.6 %) (2). Observations suggest that the presence of Baker’s cysts in knees with chronic osteoarthritic pain is associated with synovial inflammation and its grade (1). Baker’s cyst can be seen up to 46% in different studies which evaluated patients with different diagnostic modalities in rheumatology clinics (2). Commonly used modalities are MRI and USG. The prevalence of Baker’s cyst was between 0% and 2% in control groups in different studies (1,5). Treatment of the underlying disorder, steroid injection to ease the pain and surgery are the options for treatment (3). 

There are numerous reports in the literature about PET/CT interpretation pitfalls. Lesions with a high concentration of inflammatory cells, such as neutrophils and activated macrophages, also show increased F-18 FDG uptake, which can be mistaken for malignancy or distant metastasis in patients with proven or suspected cancer (6). 

Although the most common site of metastasis is the regional lymph nodes, and the distant metastases, elsewhere in the body are less common, we know that melanoma can occur in almost any organ of the body (7). Therefore, a lesion which accumulates F-18 FDG can be misinterpreted as distant metastasis in a melanoma patient. However; our knowledge, which is synovial inflammation and inflammatory joint disease are common pathological findings in Baker’s cyst, makes interpretation easier.
